# Impact of slice thickness, pixel size, and CT dose on the performance of automatic contouring algorithms

**DOI:** 10.1002/acm2.13207

**Published:** 2021-03-29

**Authors:** Kai Huang, Dong Joo Rhee, Rachel Ger, Rick Layman, Jinzhong Yang, Carlos E. Cardenas, Laurence E. Court

**Affiliations:** ^1^ The University of Texas MD Anderson Cancer Center UTHealth Graduate School of Biomedical Sciences Houston TX USA; ^2^ Department of Radiation Physics The University of Texas MD Anderson Cancer Center Houston TX USA; ^3^ Department of Imaging Physics The University of Texas MD Anderson Cancer Center Houston TX USA; ^4^ Present address: Radiation Oncology Department Mayo Clinic Phoenix AZ USA

**Keywords:** atlas‐based segmentation, auto‐contouring, convolutional neural network, CT parameters

## Abstract

**Purpose:**

To investigate the impact of computed tomography (CT) image acquisition and reconstruction parameters, including slice thickness, pixel size, and dose, on automatic contouring algorithms.

**Methods:**

Eleven scans from patients with head‐and‐neck cancer were reconstructed with varying slice thicknesses and pixel sizes. CT dose was varied by adding noise using low‐dose simulation software. The impact of these imaging parameters on two in‐house auto‐contouring algorithms, one convolutional neural network (CNN)‐based and one multiatlas‐based system (MACS) was investigated for 183 reconstructed scans. For each algorithm, auto‐contours for organs‐at‐risk were compared with auto‐contours from scans with 3 mm slice thickness, 0.977 mm pixel size, and 100% CT dose using Dice similarity coefficient (DSC), Hausdorff distance (HD), and mean surface distance (MSD).

**Results:**

Increasing the slice thickness from baseline value of 3 mm gave a progressive reduction in DSC and an increase in HD and MSD on average for all structures. Reducing the CT dose only had a relatively minimal effect on DSC and HD. The rate of change with respect to dose for both auto‐contouring methods is approximately 0. Changes in pixel size had a small effect on DSC and HD for CNN‐based auto‐contouring with differences in DSC being within 0.07. Small structures had larger deviations from the baseline values than large structures for DSC. The relative differences in HD and MSD between the large and small structures were small.

**Conclusions:**

Auto‐contours can deviate substantially with changes in CT acquisition and reconstruction parameters, especially slice thickness and pixel size. The CNN was less sensitive to changes in pixel size, and dose levels than the MACS. The results contraindicated more restrictive values for the parameters should be used than a typical imaging protocol for head‐and‐neck.

## 1 INTRODUCTION

The advancement of radiation treatment techniques has allowed precise delivery of radiation to a target with minimal toxicity to normal tissue. A crucial step in achieving precise treatment delivery is the accurate delineation of normal structures. This delineation process is prone to inter‐ and intraobserver variabilities.^1^, ^2^ This could contribute substantially to the amount of variation in patient planning using advanced radiation techniques, such as intensity‐modulated radiation therapy, which strive for subcentimeter accuracy.^3^ Furthermore, manual delineation is time‐consuming, and studies have shown that experts need at least 60 min to manually delineate the targets and organs‐at‐risk structures for an average patient with head‐and‐neck cancer.^4^ Delineation is expected to take longer if disease stage‐specific modifications are taken into consideration.

There has been much research aimed at reducing the overhead time and producing more consistent contours.^5^ There are two main categories of automatic segmentation systems for normal tissues: atlas‐based and deep learning‐based segmentation. One example of an atlas‐based tool, the multiatlas contouring system (MACS), has been clinically used in our institution for several years.^6^, ^7^, ^8^, ^9^, ^10^ More recently, deep learning‐based algorithms have been found to outperform atlas‐based algorithms in contouring some normal structures from CT images.^5^, ^11^, ^12^, ^13^ In order to take advantage of these advances, Rhee et al.^14^ recently developed an auto‐contouring algorithm for head‐and‐neck normal structures using convolutional neutral networks (CNNs). Both of these tools (MACS and CNN‐based algorithms) are expected to be deployed as part of the radiation planning assistant (RPA) system. The RPA system aims to automate the entire treatment planning process including contouring, planning, and dose calculation with minimal user interactions.^15^ In this framework, these tools will likely be used with CT images from a wide range of CT scanners, using a wide range of imaging protocols.

It is known that CT image quality affects the performance of expert contour delineations.^16^, ^17^ Thus, the performance of auto‐contour algorithms could also depend on the quality of CT images. However, to the best of our knowledge, the impact of CT image quality on auto‐contouring algorithm performance has not been assessed. The CT acquisition and reconstruction parameters investigated are slice thickness, pixel size, and CT dose. Previous studies from Ger et al. and Berthelet et al. have shown quantitative image analyses such as radiomics analysis and segmentation are impacted by different choice in slice thickness, pixel size, and CT dose.^18^, ^19^ The purpose of this study is to provide reasonable expectations of the deviations in the contours produced by two independent auto‐contouring systems providing specific CT acquisition and reconstruction parameters.

## 2 MATERIALS AND METHODS

### 2.A Image datasets

This study was performed retrospectively and did not directly involve and pose risk to study participants. The study was compliant with health insurance portability and accountability act and approved by institutional review board under protocol #PA6‐0379. This study used CT sinograms from 11 patients with head‐and‐neck cancer. Six of the CT sinograms were acquired at a Philips Brilliance Big Bore scanner with 16 × 0.75 mm slice collimation and reconstructed at the console using a range of reconstruction settings (Philips Healthcare, Netherlands). The other five patient scans were acquired at a Siemens Somatom Definition Flash scanner with 64 × 0.6 mm slice collimation and reconstructed using proprietary software ReconCT (v14.1.0.30238, Siemens Healthineers, Forchheim, Germany). Siemens scans with lower doses were simulated by adding noise in the raw projection domain prior to reconstruction using ReconCT. The methodology has been previously described and validated.^20^, ^21^, ^22^

Computed tomography scans were reconstructed from the sinograms with varying slice thicknesses, pixel sizes, and simulated mAs ranged from 10% to 100% of the original dose level. The slice spacing for all the reconstructed scans had the same value as the slice thickness. The number of patients and CT scans used for each parameter evaluation is summarized in Table 1 (both sets of data). The number of CT scans was not a multiple of the number of patients, because different options were offered by console and ReconCT at the time of reconstruction for each parameter. The range of CTDI_vol_ 16 cm for the scans was 48.1 to 61.1 mGy and tube voltage was 120 kVp. In our institution, protocols are designed such that difference from our scanners is minimized for radiological purposes. The standard clinical soft tissue kernel was applied during reconstruction, specifically, Siemens used SAFIRE‐Standard iterative reconstruction with J30S kernel and strength 3, and Philips used B or UB. UB is a slightly sharper kernel than B but it would not have a significant effect on soft tissues. Our experience, based on creating, testing and using auto‐contouring models with images from multiple scanners is that differences in scanners do not noticeably affect the contouring.

**Table 1:** The number of patients, CT scans and the levels evaluated for each parameter. Due to the availability of levels offered by console and ReconCT, the number of CT scans is not a multiple of the number of patients.

	Number of		Levels evaluated for each parameter
	Patients	CT scans	
Slice thickness	11	105	0.6, 0.75, 0.9, 1, 1.5, 1.8, 2, 3, 4, 5, 6, 7, 8, 10 mm
Pixel size	10	53	0.49, 0.59, 0.68, 0.78, 0.88, 0.98, 1.07, 1.17 mm
Dose	5	25	10%, 25%, 50%, 75%, 100% of original dose
	Total	183	

### 2.B Automated contouring

Normal tissues on all images were contoured using two approaches, MACS and a CNN‐based auto‐contouring tool. MACS used a set of 12 CT scans as atlases with structures carefully delineated and independently validated by physicians.^6^ All of the atlases used typical clinical protocols for head and neck with either 2.5 or 3 mm slice thickness, 0.98 to 1.17 mm pixel spacing, and 22.8 to 59.4 mGy CTDI_vol_ 16 cm. These values were within the investigated range for each parameter evaluated in this study. For MACS, CT is registered with each atlas using a dual‐force “Demons” deformable registration algorithm proposed.^23^ The resulting deformation vector fields are then used to deform the contours from each atlas to the new CT scan, creating multiple segmentations of one structure. These multiple segmentations, corresponding to each atlas, are then fused to produce the final contours on the input CT scan using a modified STAPLE algorithm.^7^

The CNN‐based tool was trained, validated, and tested on CT scans from 3495 patients with head‐and‐neck cancers. For all of the CT scans used for training the CNN‐based tool, the pixel spacing ranged from 0.53 to 1.37 mm, the slice thickness ranged from 1 to 3 mm, and CTDI_vol_ 16 cm ranged from 5.8 to 72.0 mGy. All of the scans used typical clinical protocols for imaging head‐and‐neck and brain tumors. The CNN‐based tool employs a two‐step process: (a) a classification model detects the existence of a structure and (b) a segmentation model delineates the detected structures in each CT slice. The classification model uses inception‐residual network (Inception‐ResNet‐v2^24^) architecture and the segmentation model uses both 3D‐VNet^25^ and a 2D fully convolutional network (FCN‐8s^26^) architecture depending on the structure size.^14^

Both the MACS and the CNN‐based tool resampled all the input data into the same voxel size of 0.98 mm × 0.98 mm × 2.5 mm with bilinear interpolation before prediction. Sixteen structures were auto‐contoured on the available images using both the MACS and the CNN‐based tools. These auto‐contours including large structures — the brain, brainstem, spinal cord, left and right eyes, mandible, and left and right parotid glands, and small structures — the left and right cochlea, esophagus, optic chiasm, left and right lenses, left and right optic nerves. Structures with predicted average volumes larger than 9 cm^3^ were classified as large.

### 2.C Contour evaluation

The normal tissue contours produced with CT images with varying reconstruction parameters were then compared against the contours produced when using clinical baseline values (i.e., baseline values standard in our clinic) with a resampling voxel of 1 mm^3^. The tool for contour comparison was validated with virtual phantoms such as boxes and spheres with known ground‐truth values. The metrics for comparisons were the Dice similarity coefficient (DSC), maximum distance Hausdorff distance (HD), and mean surface distance (MSD).^27^ Specifically for investigating the effects of slice thickness variations, auto‐contours from scans with varied slice thickness were compared with the corresponding auto‐contours from scans with 3 mm slice thickness. The slice thickness of 3 mm is the aforementioned baseline value. Similarly for pixel size, baseline value was 0.977 mm. All of the contours from CT images with added noise were compared to the contours from the original CT images with a 100% dose level. The baseline values were selected based on most common clinical values for each parameter used at our institution. The evaluated ranges were the full range of values provided by the ReconCT software and the imaging console for the Philips scanner. Similar values were reported by Kisling et al based on a survey made to the medical physics community.^28^

## 3 RESULTS

**Fig. 1:**
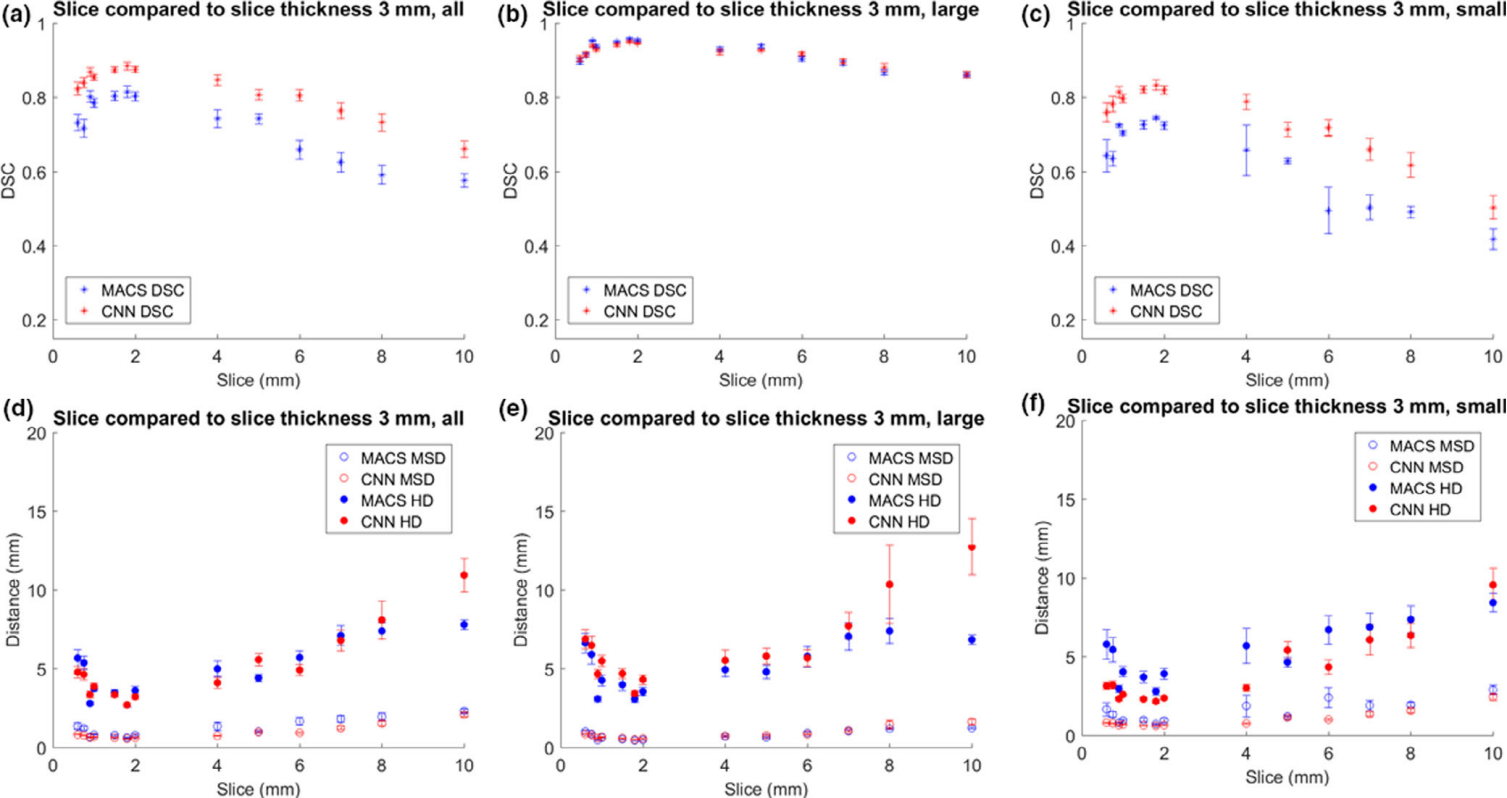
Comparison of DSC, MSD, and HD for the auto‐contours produced for varying slice thickness and those from a control of slice thickness of 3 mm. Figure parts (a) and (d) show the average values of all patients and structures. Figure parts (b) and (e) show the average values of all patients and large structures. Figure parts (c) and (f) show the average values of all patients and small structures. Large structures included the brain, brainstem, spinal cord, left and right eyes, mandible, and left and right parotid glands, and small structures included the left and right cochlea, esophagus, optic chiasm, left and right lenses, left and right optic nerves.

**Fig. 2:**
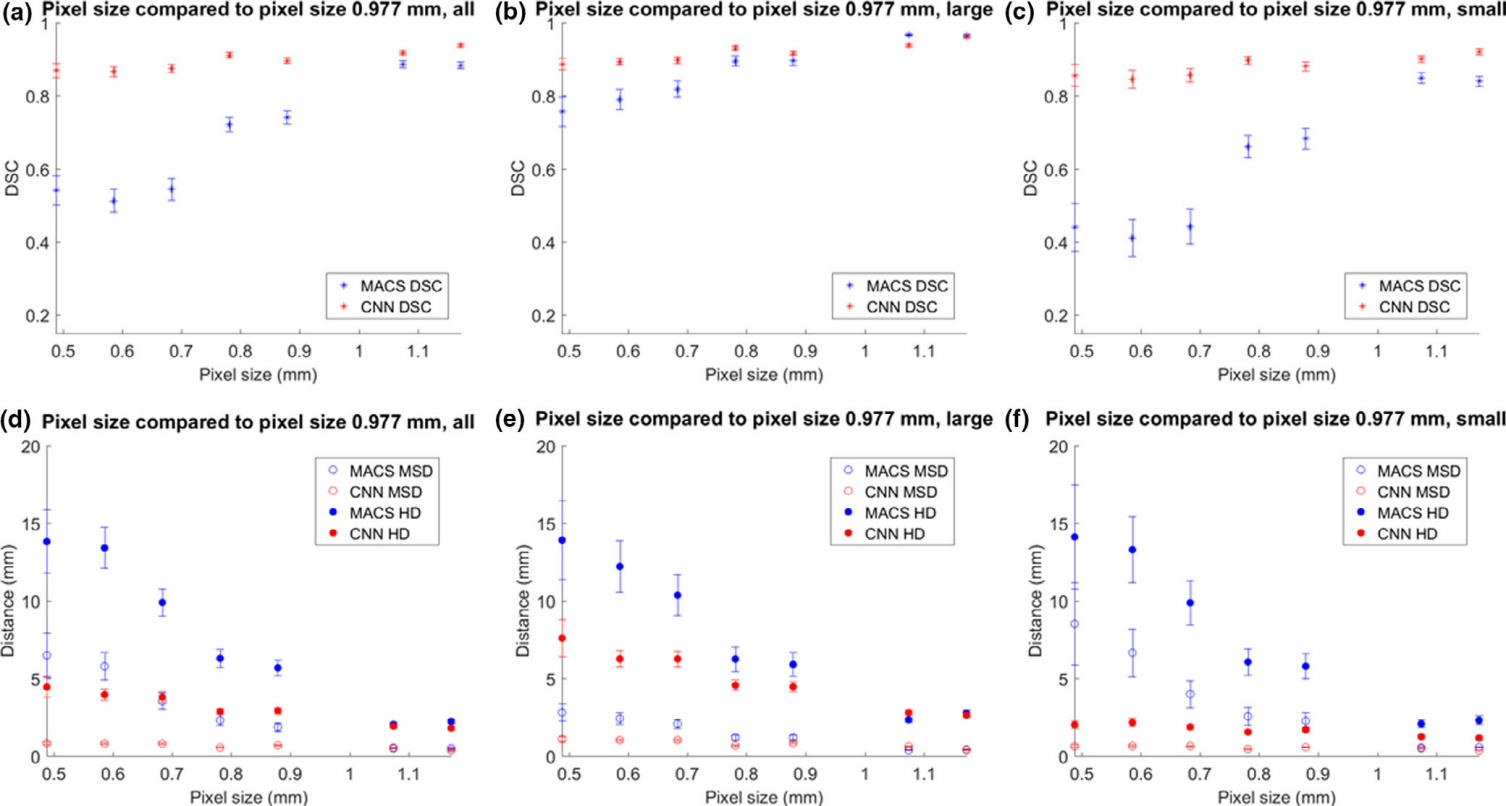
Comparison of DSC, MSD, and HD for the auto‐contours produced for varying pixel thickness and those from a control of pixel thickness 0.977 mm. Figure parts (a) and (d) show the average values of all patients and structures. Figure parts (b) and (e) show the average values of all patients and large structures. Figure parts (c) and (f) show the average values of all patients and small structures. Large structures included the brain, brainstem, spinal cord, left and right eyes, mandible, and left and right parotid glands, and small structures included the left and right cochlea, esophagus, optic chiasm, left and right lenses, left and right optic nerves.

**Fig. 3:**
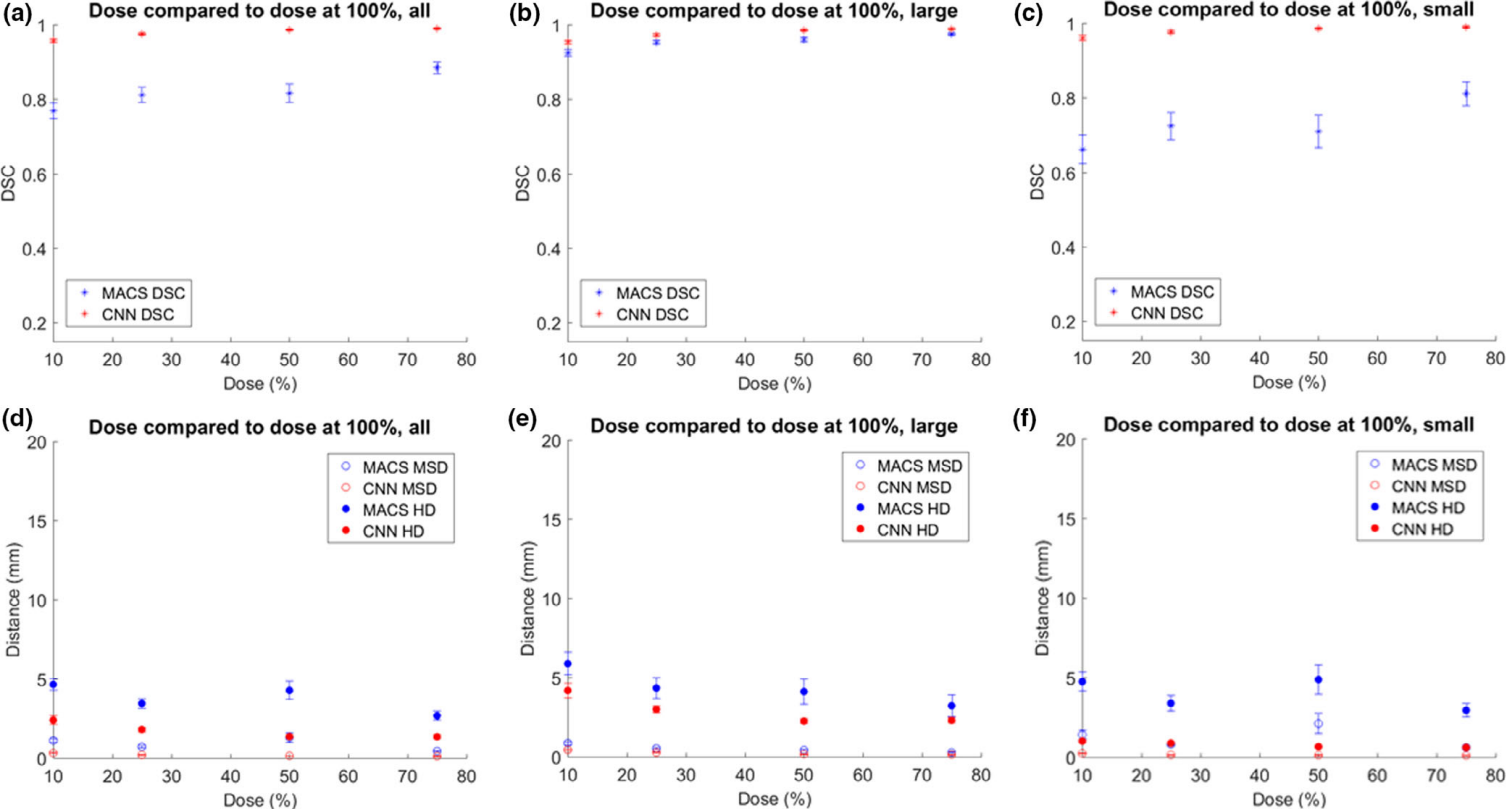
Comparison of DSC, MSD, and HD for the auto‐contours produced for varying dose and those from a control of 100% of original dose. Figure parts (a) and (d) show the average values of all patients and structures. Figure parts (b) and (e) show the average values of all patients and large structures. Figure parts (c) and (f) show the average values of all patients and small structures. Large structures included the brain, brainstem, spinal cord, left and right eyes, mandible, and left and right parotid glands, and small structures included the left and right cochlea, esophagus, optic chiasm, left and right lenses, left and right optic nerves.

The MACS and CNN‐based tool took approximately 26 and 3 min per scan, respectively, to produce contours. Figures 1, 2, 3 show the mean DSC, MSD, and HD for auto‐contours produced when varying parameter values, with the results shown separately for large, small, and all structures. The figures showed results for scans with parameter values smaller and larger than the baseline values, leaving the lines disconnected at the baseline values. This was because the contours from scans for baseline values compared to themselves had DSC of 1 and distance measures of 0 mm. Connecting the lines through perfect DSC and distance measures would create artificial peaks in figures, thus the regions were left void.

Table 2 shows the slope of a linear fit for averaged values with all structures before and after baseline. The corresponding values were plotted in (a) and (b) subfigures of Figs. 1, 2, 3. The baseline values (perfect DSC and distance measures) were not included in calculating the slope of the linear fit. Based on the slopes, the deviations in dose were smallest amongst the three parameters. Overall, the rates of change for DSC, HD, and MSD were higher for MACS than those for CNN, except for distance measures from slice thickness parameter, in which case the slopes were comparable.

**Table 2:** The slope of a linear fit for averaged values for all structures smaller and larger than baseline. There were only two data points for calculating the slope for pixel size larger than baseline.

		Pixel size		Slice thickness		Dose
		Smaller than baseline	Larger than baseline	Smaller than baseline	Larger than baseline	Smaller than baseline
MSD	MACS	−1.301	0.046	−0.026	0.020	−0.001
	CNN	−0.056	−0.124	−0.014	0.022	0.000
HD	MACS	−2.396	0.161	−0.110	0.059	−0.002
	CNN	−0.426	−0.118	−0.116	0.111	−0.002
Dice	MACS	0.623	−0.110	0.037	−0.032	0.002
	CNN	0.103	0.131	0.031	−0.030	0.000

## 4 DISCUSSION

In this study, we investigated two in‐house algorithms: a CNN‐based and a MACS‐based auto‐contouring tools. The exact impact of image resolution on an individual auto‐contouring tool might be subject to details of implementation. Overall, for the specific implementation in this study, we have demonstrated that auto‐contours can deviate with changes in CT acquisition and reconstruction parameters, especially slice thickness and pixel size. The changes in DSC were more stable for large structure than small structures.

A deviation from the perfect score (in Figs. 1, 2, 3) should be carefully interpreted. In this study, the ground truth was the results as auto‐contoured on the CT images with standard image parameters (pixel size, etc.). For example, we may expect to see an improvement in contouring quality as slice thickness is reduced, but this may not be realized in the current study because of the use of the 3 mm slice data as the baseline, which may not be perfect. The values presented in this study demonstrated the amount of deviation from the results of baseline, and were not necessarily indicative of improvements or deteriorations in performance compared with the results of baseline. In future work digital phantoms with a known ground truth could be developed and evaluated.

Both MACS and CNN resampled input data to a fixed resolution. For MACS, input data would be resampled to the resolution of atlases. Therefore, input data with much finer resolution did not have pronounced advantage as the information would be lost in the resampling step. However, even with the same resolution, the resulting data slices may shift due to different implementations of where the image origin should be. This might contribute in part to the deviation for metrics in the figures due to image resolution.

Additionally, there were uncertainties associated with the contour comparison tool. These uncertainties mainly stem from the volume calculation especially at the beginning and tailing slices of the structures. Therefore, by setting the reference voxel to a standard of 1 mm^3^, the impact of uncertainties from the comparison tool to the final results was minimal.

Specifically, for slice thickness, large structures could achieve DSC values of more than 80%. Overall, small structures had lower DSC values compared to large structures. Additionally, the trend was amplified in slice thicknesses larger than 3 mm compared to smaller. For head‐and‐neck CT simulations, slice thickness is recommended to be no more than 3 mm.^29^, ^30^ A reference protocol for head CT from manufacturer can have slice thickness range from 0.5 to 6 mm depending on the machine specifications.^31^ Both results for DSC and distance measures indicated larger deviations when the slice thickness is larger than 6 mm. The variation for the slice thickness smaller than 3 mm (0.6–3 mm) may be explained in part by image resampling within the auto‐contouring algorithm. The slight increase in trend as the slice thickness decreases from 3 to 0.6 mm could result from missing slices in the most cranial and caudate parts of the structures. The variation for values smaller than 3 mm is more stable than the upward trend for values larger than 3 mm. Overall, the results did not indicate a more restrictive value for slice thickness to be implemented than what has been recommended by guidelines.

In regards to pixel size variations, CNN‐based solution produced more stable results compared to the corresponding values from MACS. When the pixel size decreases, the images become noisier and the interpolation in resampling process might be negatively impacted causing deteriorated predictions. The amplified response of MACS compared to CNN due to small pixel sizes may be partly caused by limited range of pixel size in the atlases. Even though all of the input data would be resampled, no atlases used a pixel size smaller than 0.98 mm, whereas CNN‐based tool was trained on a much wider range of pixel sizes (from 0.53 to 1.37 mm). Even with the same resolution, more variations in the training data might have contributed to the relatively more robust result from CNN‐based tool. Further studies with varying standard resampling grid in the auto‐contouring tools are required to systematically evaluate the impact of resampling on contour results.

As for changes in dose levels, CNN‐based solution was less sensitive to the change in dose compared to MACS. This may in part due to the wider range of CTDI_vol_ values in the training dataset for CNN‐based tool. The decrease in dose affects DSC values of the small structures more than the large structures, especially for MACS. In terms of distance measures, both HD and MSD increased as dose decreases. The HD values from CNN for large structures were larger than those for the smaller structures. This may be explained by postprocessing in CNN algorithm would only retain the largest predicted volume if there are more than one volume predicted for one structure. If there were discontinuities in predictions, postprocessing would remove smaller volumes, making predicted volumes much smaller than the other scans for comparison. The details of the postprocessing in algorithms may vary considerably based on the specific implementations.

For CNN‐based method, small and large structures used different architectures. For the atlas‐based method, both large and small structures used the same set of atlases and registration process. It is possible some of the differences in the evaluation results for CNN‐based method may be attributed to the segmentation networks used. However, the results from MACS‐based method follow the same trend and are amplified compared to the corresponding data from CNN‐based method. Therefore, it is reasonable to conclude the contribution of different networks to the discrepancies between large and small structures are minimal. One of the limitations of this study is the only two specific auto‐contouring algorithms were investigated. These two algorithms have been implemented clinically in our institution. However, the specific details of implementation vary between various auto‐contouring tools. Thus, the results of this study may not be generalized to other auto‐contouring tools without further investigation.

## 5 CONCLUSIONS

We investigated the impact of CT image quality parameters including slice thickness, pixel size, and dose levels, on auto‐contouring algorithms. The results showed that CNN‐based tool was less impacted by the change of pixel size and dose levels as compared to MACS. The results do not indicate more restrictive values for the image resolutions should be used than a typical imaging protocol for head‐and‐neck. The assessment of the impact of CT image quality parameters is warranted before commissioning auto‐contouring algorithms for clinical uses.

## CONFLICT OF INTEREST

Other aspects of the Radiation Planning Assistant project are funded by Varian Medical Systems.

## CONTRIBUTION STATEMENTS

All authors contribute substantially to the conception, data acquisition, analysis, and writing of the manuscript.
